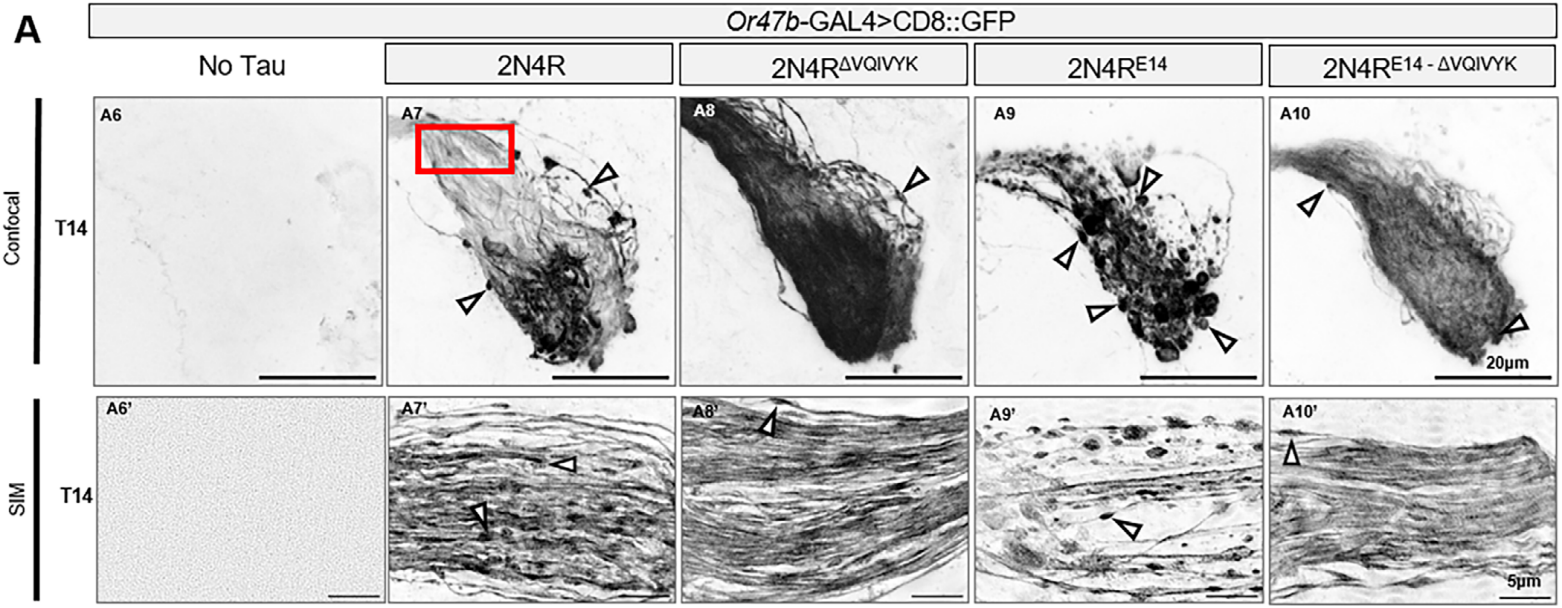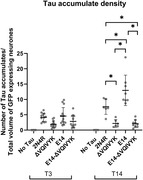# Aggregation Trumps Phosphorylation in Tau‐mediated Toxicity

**DOI:** 10.1002/alz.094727

**Published:** 2025-01-09

**Authors:** Amber Cooper, Brad Richardson, Yongrui Zhang, Miguel Ramírez Moreno, Ben Batchelor, Ray Price, Efthimios M. C. Skoulakis, Douglas Allan, Amritpal Mudher

**Affiliations:** ^1^ University of Southampton, Southampton United Kingdom; ^2^ Biomedical Sciences Research Centre "Alexander Fleming”, Athens Greece; ^3^ University of British Columbia, Vancouver, BC Canada

## Abstract

**Background:**

Disease‐modifying therapies for tauopathies like Alzheimer’s disease have targeted Tau hyperphosphorylation and aggregation, as both pathological changes are implicated in Tau‐mediated toxicity. However, the interplay between Tau hyperphosphorylation and aggregation, and their relative contribution towards overall Tau toxicity is not entirely understood.

**Method:**

Leveraging the genetic tractability of *Drosophila*, we generated novel transgenic lines designed to manipulate Tau phosphorylation and aggregation to address these questions and unpick the relationship between these pathological changes. Tau accumulation and neurodegenerative phenotypes were visualised and quantified using Confocal and super resolution (Structured Illumination Microscopy, SIM) microscopy. Changes in total and phosphorylated Tau in *Drosophila* brain homogenates were also quantified by western blot analysis.

**Result:**

We report profound neurodegeneration following expression of phospho‐mimicking human Tau, indicative that phosphorylation is a key driver of Tau toxicity in tauopathies. However, when Tau is rendered unable to aggregate, through deletion of a known aggregation motif, Tau toxicity was completely abolished even in the presence of phospho‐mimicking mutations. To phenocopy the neuroprotective effects of genetically suppressing aggregation, a peptide inhibitor that targets the same aggregation‐promoting motif, was also efficacious in significantly reducing Tau phenotypes throughout the lifespan of Tau expressing flies.

**Conclusion:**

Our data demonstrates that Tau phosphorylation mediates neurodegeneration through a mechanism gated via Tau aggregation such that if hyperphosphorylated Tau cannot aggregate, it is no longer toxic. These results should inform the next generation of Tau therapeutics as suppression of Tau aggregation may have profound disease‐modifying potential without the need to clear pathogenic phosphorylated species. Funded by Alzheimer’s Society of Canada and Mike & Valeria Rosenbloom Foundation Research Award: Award Number: AWD‐019542 ALZSOCCA 2020o.